# Longitudinal changes in personal wellbeing in a cohort of people who inject drugs

**DOI:** 10.1371/journal.pone.0178474

**Published:** 2017-05-31

**Authors:** Nick Scott, Elise R. Carrotte, Peter Higgs, Mark A. Stoové, Campbell K. Aitken, Paul M. Dietze

**Affiliations:** 1 Burnet Institute, Melbourne, Australia; 2 Department of Epidemiology and Preventive Medicine, Monash University, Melbourne, Australia; 3 National Drug Research Institute, Faculty of Health Sciences, Curtin University, Melbourne, Australia; 4 Department of Public Health, La Trobe University, Bundoora, Australia; University of New South Wales, AUSTRALIA

## Abstract

**Aims:**

To determine whether the self-reported personal wellbeing of a cohort of people who inject drugs (PWID) changes over time, and to identify longitudinal correlates of change.

**Methods:**

We used Personal Wellbeing Index (PWI) scores reported between April 2008 and February 2015 by 757 PWID (66% male) enrolled in the Melbourne Injecting Drug Use Cohort Study (2,862 interviews; up to seven follow-up waves). A mixed-effects model was used to identify correlations between changes in temporal variables and changes in individual PWI scores while controlling for demographic variables.

**Results:**

The cohort’s mean PWI score did not significantly differ over time (between 54.4/100 and 56.7/100 across the first four interview waves), and was 25–28% lower than general Australian population scores (76.0/100). However, there were large variations in individuals’ PWI scores between interviews. Increased psychological distress, moving into unstable accommodation, reporting intentional overdose in the past 12 months and being the victim of assault in the past six months were associated with declines in PWI scores.

**Conclusions:**

Participants experienced substantially lower levels of personal wellbeing than the general Australian population, influenced by experiences of psychological distress, assault, overdose and harms related to low socioeconomic status. The results of this study suggest a need to ensure referral to appropriate housing and health support services for PWID.

## Introduction

People who inject drugs (PWID) experience lower health-related quality of life than the general population [1]. Cross-sectional research has reported a range of correlates of low quality of life in this population, including older age [2], lower educational level [3], unemployment [4], opioid overdose [5, 6], mental illness [5, 7–9], history of imprisonment [6, 10], insecure housing [10], being assaulted [4, 10], younger age at first injection [4], not receiving opioid substitution therapy [6] and alcohol and other substance use [3, 11, 12]. However, most previous research uses quality of life measures that focus on physical health, commonly measured through a symptom checklist, which is only one component of quality of life [13, 14]. A more holistic and theoretically sound way to examine quality of life is subjective personal wellbeing, which focuses on wellbeing across several dimensions, including an individual’s personal satisfaction with their health, life achievement, personal relationships and life as a whole [15].

The self-reported personal wellbeing of a sample of Australian PWID was examined by Dietze et al. [16], who found significantly lower scores on the Personal Wellbeing Index (PWI) [17] in this group than the Australian general population, with lower scores associated with unemployment, past six-month serious mental health problems and higher frequency of injecting. However, this cross-sectional analysis was limited in its ability to detect changes in PWI scores in response to environmental stressors, life events or interventions. For example, it is possible that although these factors identify groups of individuals who experience poor personal wellbeing, they may not predict a significant change in personal wellbeing over time. Longitudinal analysis is required to detect these associations [18].

The PWI has previously been used longitudinally across various studies, including studies of spinal cord injury [19], gambling addiction [20] and body image in the general population [21]; however, to our knowledge no longitudinal studies have been conducted using the PWI as an outcome measure with community-based PWID. Previous international longitudinal studies have examined quality of life before and after drug treatment commencement, usually with the World Health Organization’s brief Quality of Life instrument, the WHOQOL-B [22–26], but are limited to opioid-dependent participants undergoing treatment. These studies have generally demonstrated improvements in quality of life after treatment. Knowledge of correlates of change in the personal wellbeing of community-based PWID would enable public health interventions to be more appropriately and effectively targeted.

This study aimed to determine whether self-reported personal wellbeing of a cohort of people who inject drugs (PWID) changes over time, and to identify longitudinal correlates of change. Previous cross-sectional research has identified a significant link between mental health and quality of life among PWID, indicating that these two concepts are closely related [7, 8, 16, 27]; however, here we identify the relationships between life events and personal wellbeing outside of their influence through psychological distress.

## Methods

### Data source

Briefly, 688 PWID were enrolled in the Melbourne Injecting Drug User Cohort Study (MIX) between April 2008 and January 2010. Participants were eligible for MIX if they reported being aged between 18 and 30 years (median at baseline 27.6 years, IQR 24.3–29.6 years) and had injected heroin or methamphetamine at least six times over the previous six months. Sixty-nine additional PWID from another cohort with similar recruitment criteria, Networks II [28], were rolled into the study in 2011 (henceforth “MIX participants” refers to the combined cohort of 757 participants, 66% of whom were male). Participants were interviewed face-to-face approximately annually to obtain detailed information about living circumstances, health and drug use history. Between 18 April 2008 and 13 February 2015, 2862 interviews had been conducted, with individuals having a maximum of seven follow-up interviews (N = 757, 584, 510, 432, 328, 217, 33, 1 for the baseline and seven follow-up waves respectively; note that individuals’ interviews were numbered successively regardless of the time between them). The median time between individuals’ successive interviews was 366 days (inter-quartile range 322–432 days, range 22–2154 days). Further details including comparisons of the original MIX and Networks II participants, interview timings and loss to follow-up are provided in the supplementary material (S1 File).

### Outcome measure

We used the PWI to measure subjective personal wellbeing. The PWI questionnaire asks participants to rate seven sub-components of their personal wellbeing—their standard of living, health, achievements in life, personal relationships, perception of safety, community involvement and future security—on an 11-point scale (0–10; 0 indicates ‘no satisfaction at all’ and 10 indicates ‘completely satisfied’). Participants are told that ‘5’ is a neutral response. Responses are summed and scaled to give final PWI scores ranging from 0 to 100, with higher scores indicating higher levels of subjective personal wellbeing. General Australian population norms are available [29]. The PWI has good psychometric properties [17] and has been used previously in research involving PWID [16]. The PWI is administered near the end of the MIX interview.

### Independent variables

Relevant MIX variables were classified as either stable or temporal. Stable variables included sex, age at baseline (<20, 20–24, 25–29, 30+ years), country of birth (Australia, other), language other than English spoken (no, yes), education (<year 10, year 10–11, year 12 or higher), length of injecting career at baseline (<3, 3–5, 6–8, 9–11, 12–14, 15+ years) and incarceration history at baseline (none, once, twice, three or more times). Temporal variables included income type (none, wage, government pension, other), employment (no, yes), accommodation type (owner-occupied, private rental, public housing, unstable), illicit drug used most in the past month (heroin, methamphetamine, cannabis, other), alcohol use as measured by scores on a variant of the Alcohol Use Disorders Identification Test Consumption (AUDIT-C) scale [30] (with scores of 0, 1–7 and > = 8 classified according to [12] as abstinent, low risk or high risk respectively); injecting drug use frequency (total reported injections, of all drugs, in the past week), inject more than usual in the past six months (no, yes), inject alone more than 80% of the time (no, yes), blood borne virus (BBV) transmission risk (Blood Borne Virus Transmission Risk Assessment Questionnaire–Short Version, (BBV-TRAQ-SV); [31] score), opioid substitution therapy status (no, yes), general practitioner (GP) attendance in the past month (no, yes), attended psychiatrist/psychologist/social worker/drug counsellor in the past month (no, yes), attended an emergency department (ED) in the past month (no, yes), heroin overdose in the past six months (no, yes), intentionally overdosed in the past 12 months (no, yes), been the victim of an assault in the past six months (no, yes) and been arrested in the past six months (no, yes).

From November 2010 onwards the Kessler Psychological Distress Scale (K10) survey, a 10-item measure of psychological distress over the past four weeks [32], was included in the MIX questionnaire. This means that the K10 has been administered in 62% (1770/2862) of interviews, with 75% (564/757) of participants having at least one recorded score. The K10 is scored between 10 and 50, with higher scores indicating higher levels of psychological distress. Scores of 27 or more among PWID are likely to indicate a current affective disorder [33] and therefore psychological distress was measured dichotomously (K10 score <27 or K10 score > = 27).

### Data analysis

#### Comparison of PWI scores across interview waves and to the general population

MIX participants’ mean PWI scores and mean sub-component scores were compared across the first four interview waves (N = 726, 570, 501 and 426 interviews respectively) and to PWI scores reported by the general Australian population in 2013 [29]. The statistical significance of time trends among MIX participants were tested in the mixed-effects regression (below). To determine the degree that individual PWI scores vary between follow-up interviews, histograms of the change in individual PWI scores between interviews were plotted for the first three transitions (baseline to follow-up 1, N = 546 transitions; follow-up 1 to follow-up 2, N = 487 transitions; and follow-up 2 to follow-up 3, N = 422 transitions).

#### Mixed-effects regression model

Associations between changes in temporal variables and changes in individuals’ PWI scores were measured using a linear mixed-effects model. Mixed-effects models allow incorporation of both time-varying as well as non-time-varying predictors and allow for values missing at random, unequally spaced time points of assessment (within and between participants) and differing numbers of time points from participant to participant [34]. Specifically, the equation estimated was:

$$y_{it}=\beta X_{it}+\delta W_{i}+{\mu +\nu }_{i}+\varepsilon_{it}$$

where for each participant *i* at interview wave *t*, *y*_*it*_ is their reported PWI score, *X*_*it*_ is a vector of time-varying variables at interview wave *t*, *β* is a vector of fixed-effect regression coefficients, *W*_*i*_ is a vector of time-invariant variables (at baseline values for participant *i*), *δ* is a vector of fixed-effect regression coefficients, *μ* is a constant, *ν*_*i*_ is a participant random-intercept to be estimated (with $\nu_{i}∼N(0,\sigma_{\nu }^{2})$) and *ε*_*it*_ is a random error term. All 1770 interviews where the K10 survey was administered were included in the model.

The 1770 interviews included in the model were conducted by 11 different interviewers (median 102 interviews each; range 1–485). To control for interviewer bias an additional variable for interviewer was included in *X*_*it*_ (not shown in our results).

To test for any statistically significant trends over time, a continuous variable “time in study” was included as a fixed-effect term in the model. This variable was calculated for each participant-interview as the time (in years) from the participant’s baseline interview.

Recent assault and intentional overdose have previously been associated with increases in psychological distress among PWID [18], so to control for this potentially mediating factor, only the subset of data where both PWI and K10 scores were available was included in the longitudinal analysis (i.e. post November 2010). However, when the analysis was repeated using the full set of interviews but without K10 as a covariate, qualitatively similar results were obtained (see S1 File).

Given the complex study population and array of potential drivers of change in PWI, all covariates were maintained in the model to attempt to control for their potential confounding effects. Statistical analysis was performed in Stata version 13.

## Results

### PWI scores compared to the general population

**Fig 1:**
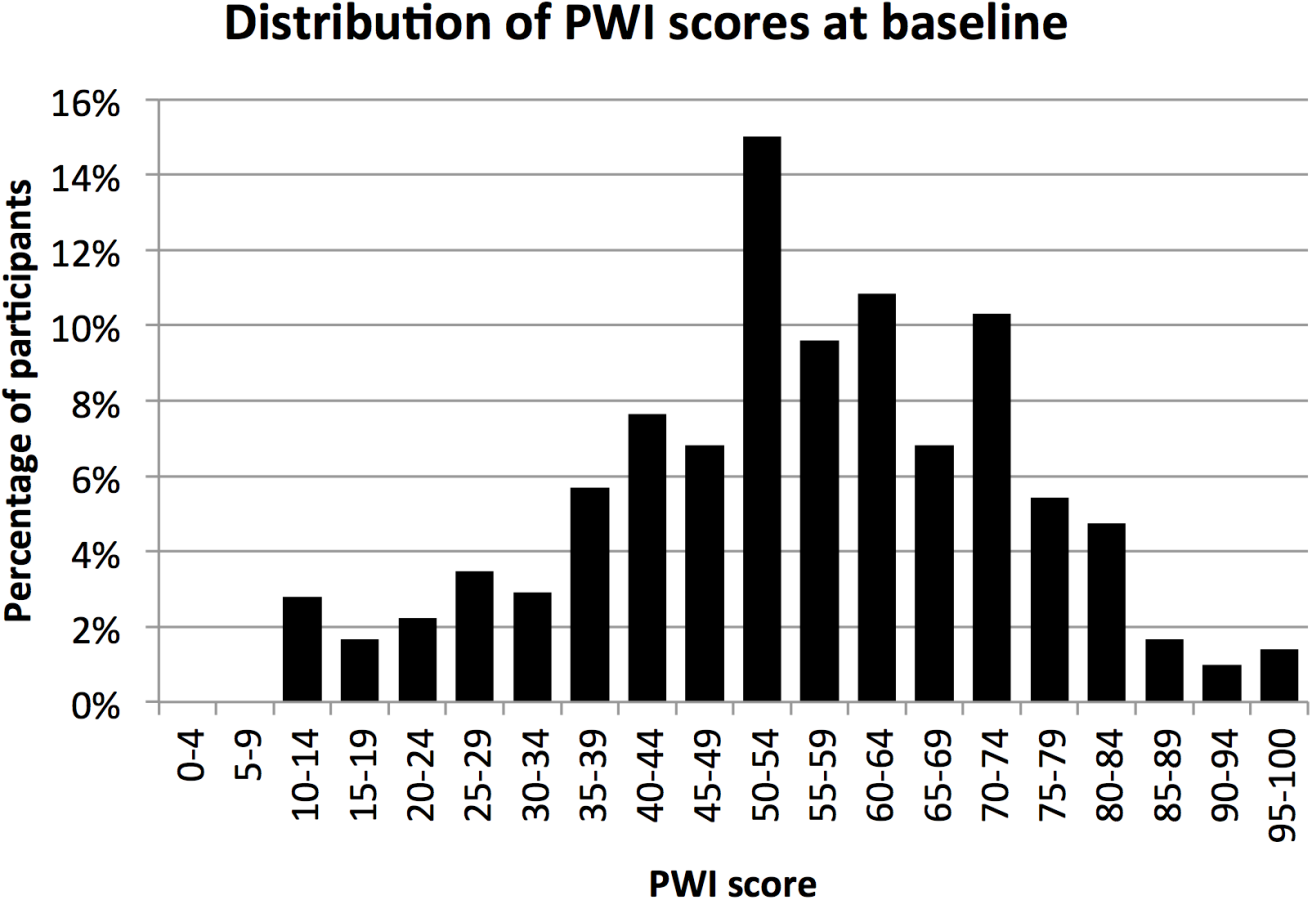
PWI scores. Distribution of PWI scores at baseline.

The mean PWI score for MIX participants at baseline was 54.9 (95%CI 53.5–56.3) (Fig 1), and there were no significant differences in PWI scores between men and women; mean PWI scores were 54.6 (95%CI 53.0–56.2) and 55.5 (95%CI 52.9–58.1) for men and women respectively.

### Variation in PWI scores over time

In each of the first four interview waves, MIX participants reported mean overall PWI scores between 25% (19.3 points) and 28% (21.1 points) lower than the general Australian population (Table 1) [29]. The mean PWI scores for each sub-scale were also substantially lower than those of the general Australian population. The greatest discrepancy between MIX participants and the general Australian population was in the ‘achievements in life’ sub-category, where MIX participants’ mean scores were between 33% (2.4 point) and 38% (2.8 points) lower. The mean ‘feelings of safety’ score for MIX participants was more than 1.5 points higher than their score in any other sub-category, yet in all interview waves this was still more than 10% (0.8 points) below the rest of the Australian population.

**Table 1:** PWI scores and component scores of the MIX cohort (baseline to follow-up three interviews) compared to the general Australian population.

		MIX scores			
	Australian population	Baseline	Follow-up 1	Follow-up 2	Follow-up 3
	*mean* (*SD*)	*mean* (*SD*)	*mean* (*SD*)	*mean* (*SD*)	*mean* (*SD*)
***N***	1972	726	570	501	426
**Overall**	76.0 (12.3)	54.9 (19.0)	56.7 (18.9)	55.8 (19.5)	56.3 (19.1)
Standard of living	7.8 (1.6)	5.2 (2.6)	5.7 (2.6)	5.6 (2.5)	5.6 (2.5)
Health	7.4 (1.9)	5.6 (2.3)	5.6 (2.3)	5.5 (2.3)	5.4 (2.3)
Achievements in life	7.3 (1.9)	4.5 (2.6)	4.9 (2.6)	4.8 (2.6)	4.8 (2.5)
Personal relationships	8.0 (2.1)	5.5 (2.7)	5.5 (2.7)	5.6 (2.7)	5.6 (2.6)
Feelings of safety	8.1 (1.7)	7.0 (2.6)	7.2 (2.6)	7.1 (2.6)	7.3 (2.4)
Feeling part of the community	7.3 (1.9)	5.2 (3.0)	5.3 (2.9)	5.1 (3.1)	5.2 (2.9)
Future security	7.2 (2.0)	5.3 (2.8)	5.4 (2.7)	5.3 (2.9)	5.4 (2.7)
Life as a whole	7.7 (1.7)	5.4 (2.5)	5.7 (2.3)	5.5 (2.5)	5.5 (2.4)

**Fig 2:**
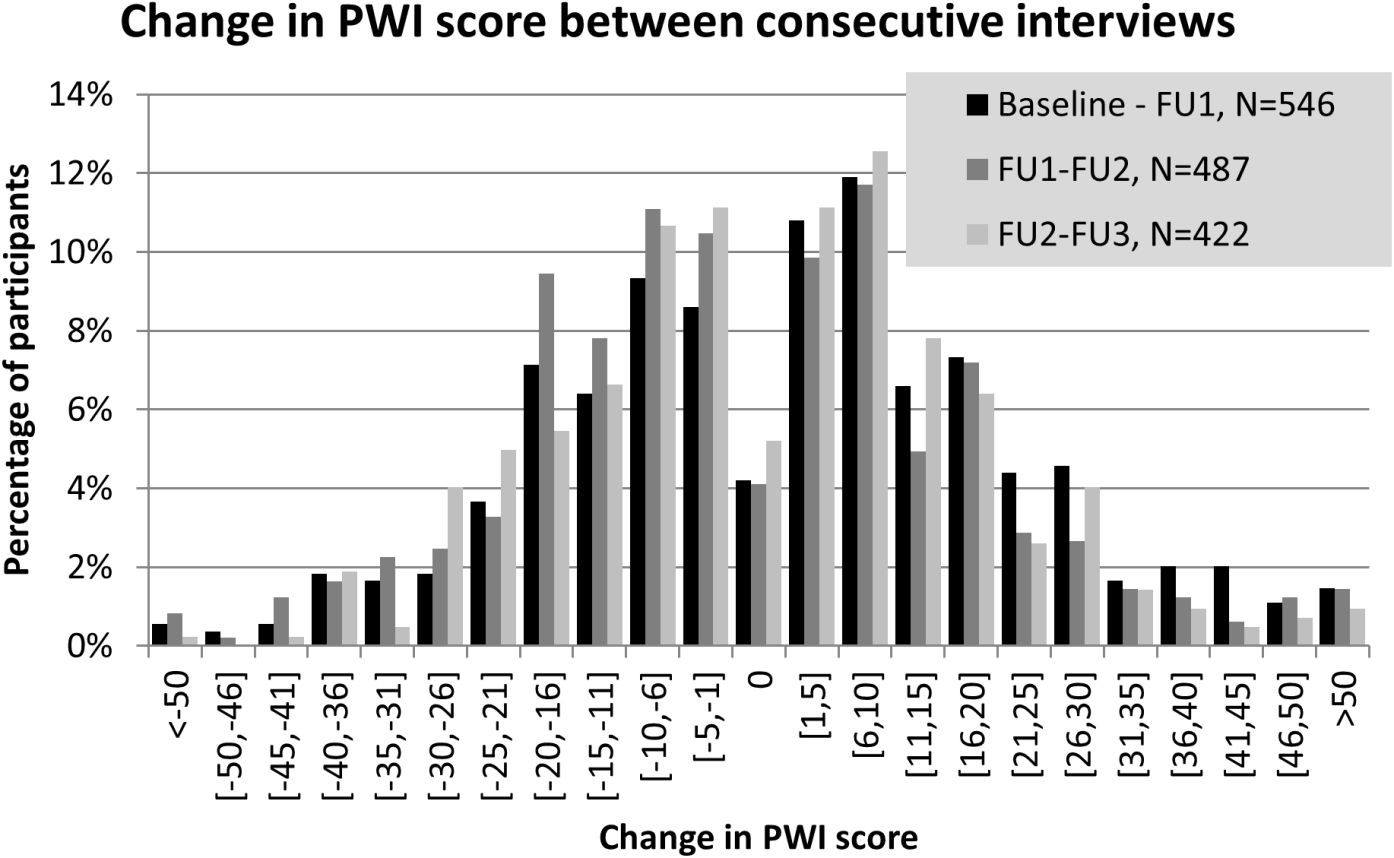
Variations in individual PWI scores. Distributions of the differences in PWI score between interviews for the first three transitions (baseline to follow-up 1, follow-up 1 to follow-up 2 and follow-up 2 to follow-up 3).

Although the cohort’s mean PWI scores varied little between interview waves, we detected considerable variation in individuals’ PWI scores (Fig 2). In interview waves 1, 2 and 3, only 24%, 24% and 27% of participants respectively had PWI scores within 5 points of their score at their previous interview, while 28%, 23% and 23% of participants respectively had scores that varied by more than 20 points from their previous interview’s score. The variation was approximately symmetric in direction, accounting for relatively stable mean PWI scores over time; 42%, 51% and 46% of participants had decreases in PWI scores, and 54%, 45%, 49% of participants had increases in PWI scores between the first three follow-up interviews respectively. Similar variation was found for PWI sub-component scores (not shown).

### Mixed-effects regression model

Table 2 shows the coefficients of the mixed-effects regression model. Some statistically significant correlations were detected among temporal variables: an increase in psychological distress, moving into unstable accommodation, injecting more than usual in the past 6 months, intentionally overdosing in the past 12 months and being the victim of assault in the past 6 months were significantly correlated with a decrease in PWI score; while becoming employed and changing drug used most in the past month from heroin to other were significantly correlated with an increase in PWI score. Consistent with Table 1, there was no statistically significant change in PWI scores over time.

**Table 2:** Linear mixed-effects regression results for PWI scores, temporal variables. Includes only MIX interviews at which both the PWI and K10 were administered.

	Mixed-effects model; N = 1,416		
	*Adjusted coefficient*	*95%CI*	*p-value*
***Temporal variables***			
K10 score > = 27 (vs. no)			
Yes	-14.92***	(-16.91, -12.93)	**<0.001**
Time in study	0.06	(-0.63, 0.74)	0.871
Main income source (vs. wage or salary)			
Government allowance	-1.16	(-4.20, 1.89)	0.457
Other	-2.06	(-6.11, 1.99)	0.318
Employed (vs. no)			
Yes	2.57*	(0.35, 4.79)	**<0.05**
Current accommodation type (vs. Owner-occupied)			
Private rental	-2.39	(-4.89, 0.11)	0.061
Public housing	-1.16	(-4.20, 1.88)	0.455
Unstable	-5.39***	(-8.47, -2.31)	**0.001**
Drug used most in the past month (vs. heroin)			
Methamphetamine	0.23	(-3.22, 3.69)	0.894
Cannabis	2.20	(-0.05, 4.45)	0.056
Other	2.87*	(0.49, 5.25)	**<0.05**
AUDIT C (vs. 0)			
1–7	-0.76	(-2.74, 1.23)	0.454
> = 8	1.16	(-1.20, 3.53)	0.336
Total injections in the past week	-0.05	(-0.14, 0.05)	0.368
Inject more than usual in the past 6 months (vs. no)			
Yes	-2.14*	(-3.97, -0.31)	**<0.05**
Use alone more than 80% of the time (vs. no)			
Yes	-0.49	(-2.18, 1.20)	0.570
BBV-TRAQ-SV score	-0.06	(-0.13, 0.00)	0.069
Currently on OST (vs. no)			
Yes	1.60	(-0.24, 3.44)	0.087
Attended a GP in the past month (vs. no)			
Yes	0.47	(-1.35, 2.29)	0.612
Any mental health assistance in the past month (vs. no)			
Yes	0.90	(-0.86, 2.65)	0.316
Attended ED in past month (vs. no)			
Yes	-0.69	(-3.35, 1.97)	0.612
Heroin overdose in the past six months (vs. no)			
Yes	-1.18	(-4.17, 1.8)	0.437
Intentional overdose in the past12 months (vs. no)			
Yes	-7.24**	(-11.65, -2.83)	**0.001**
Assault victim in past six months (vs. no)			
Yes	-3.66***	(-5.63, -1.69)	**<0.001**
Arrested in the past 12 months (vs. no)			
Yes	0.23	(-1.54, 1.99)	0.801
***Stable variables***			
Sex (vs. female)			
Male	-1.43	(-3.91, 1.04)	0.257
Age at interview	-0.10	(-0.31, 0.10)	0.323
Recruitment site (vs. Inner West)			
Central	2.26	(-1.68, 6.20)	0.261
Outer-Urban	2.36	(-1.74, 6.45)	0.259
Country of birth (vs. outside of Australia)			
Australia	0.71	(-2.63, 4.05)	0.676
Speak a language other than English (vs. no)			
Yes	0.21	(-3.85, 4.28)	0.918
Education (vs. <year 10)			
Year 10–11	2.63	(-0.41, 5.66)	0.090
Year 12 or higher	3.12*	(0.12, 6.13)	**<0.05**
Duration of injecting career (years)	0.01	(0.00, 0.01)	0.209
Incarceration history (vs. never)			
Once	-1.04	(-3.92, 1.83)	0.478
Twice	0.68	(-2.59, 3.95)	0.682
Three or more times	-0.06	(-3.16, 3.03)	0.968
***Constant***	63.68***	(52.99, 74.38)	**<0.001**
***Participant random-effect term***			
Standard deviation	10.20	(9.09, 11.45)	
***Residual (error term)***			
Standard deviation	12.09	(11.43, 12.79)	

*p < 0.05

**p < 0.01

***p < 0.001

Among stable variables, PWI scores were found to be statistically significantly higher for participants that had a year 12 or higher education than participants who had < year 10 education (Table 2).

## Discussion

We used longitudinal analysis to identify correlates of change in the personal wellbeing of a community-based cohort of PWID. We found that participants experienced substantially lower levels of personal wellbeing than the general Australian population, and changes in individuals’ PWI scores were correlated with recent experiences of psychological distress, assault, overdose and harms related to low socioeconomic status.

For all of the PWI subscales except for personal safety, average scores reported by participants at each interview wave were below 6/10, the lower limit for ‘healthy’ ratings [35]. Low scores across all subscales highlight that participants’ relatively poor quality of life relates to many aspects of their lives, and emphasises a need to focus interventions on all areas of personal wellbeing for PWID: standard of living, health, achievements in life, personal relationships, feeling part of the community, and future security. These results also indicate a need to consider these domains in the drug treatment of PWID, as is being done in the Australian Treatment Outcomes Profile (ATOP) study [36].

Mean PWI and PWI subscale scores were remarkably consistent across interview waves; however, the consistency in mean scores masked large variations in individuals’ PWI scores that were associated with environmental stressors and health indicators. The extent of individual variation suggests that the PWI is sufficiently sensitive to identify factors associated with changes in personal wellbeing among PWID, and highlights the importance of using longitudinal data to detect variables associated with changes in PWI scores.

Increases in psychological distress were associated with declines in PWI scores. This is not surprising, since psychological distress is a core component of personal wellbeing and the two measures have previously been found to be correlated [7, 8, 16, 27]. Psychological distress among MIX participants has previously been correlated with intentionally overdosing and being assaulted [18], meaning that there may have been some collinearity present in this analysis (see Table A in S1 File). However, our results indicate that these events are associated with PWI scores independent of psychological distress, and are therefore likely to impact on personal wellbeing across domains other than mental wellbeing.

Identifying correlates of change in PWI scores provides important insight as it characterizes areas that, if targeted by interventions, can lead to significant improvements in personal wellbeing. For example, to have the greatest impact on the personal wellbeing of PWID, priority interventions should include support services for individuals who have experienced or are at risk of experiencing assault or psychological distress, and housing services to prevent PWID from living in unstable accommodation.

Our study is limited by several factors. First, our sample was recruited from across the city of Melbourne and may not be generalisable to all PWID. Second, comparison between regression results using the full dataset and data collected from November 2010 onwards (when K10 scores became available), as seen in our supplementary material (Table A in S1 File), indicates that are some variables (e.g. BBV-TRAQ score, attending an ED in the past month, drug used most, age at first interview and education level) may be influenced by loss to follow-up in the cohort or affected by regression to the mean as a result of recruitment bias. This may limit the power of our analysis. Third, we have not disaggregated the direction of associations in the longitudinal model; for example, the association between change in accommodation type and change in PWI scores was calculated from a combination of 1) people moving into unstable accommodation and reporting decreased PWI scores; and 2) people moving out of unstable accommodation and reporting increased PWI scores. Distinguishing these correlations would spread data more sparsely, limiting statistical power, and in general provide little additional insight. Instead, we have elected to be mindful of our interpretations.

## Conclusions

Consistent with previous work, our results show that PWID experience substantially lower levels of personal wellbeing than the general Australian population across all sub-scales of the PWI. However, we have shown that personal wellbeing changed dramatically over time for most cohort members, and our measurement was sensitive to certain events that characterise the lives of PWID. Considering the low levels of personal wellbeing in this sample, there is a need to continue efforts to improve personal wellbeing among PWID that are cognisant of the effects of temporal negative life events such as assault and unstable housing. The results of this study suggest a need to ensure referral to appropriate housing and health support services for PWID.

## Supporting information

S1 File Supplement. Additional analysis of PWI and K10 interactions; descriptions of the Melbourne Injecting Drug User Cohort Study (MIX) and Networks II study; combined cohort characteristics at each interview wave; and details of loss to follow-up.
(DOCX)
