# Supplementary material for: Longitudinal changes in personal wellbeing in a cohort of people who inject drugs
Source: PLoS One. 2017 May 31;12(5):e0178474. doi: 10.1371/journal.pone.0178474 (PMC5451053; doi:10.1371/journal.pone.0178474)
Supplement: S1 File — Additional analysis of PWI and K10 interactions; descriptions of the Melbourne Injecting Drug User Cohort Study (MIX) and Networks II study; combined cohort characteristics at each interview wave; and details of loss to follow-up. (DOCX) [file pone.0178474.s001.docx]

**Supplementary material**

**Additional analysis of PWI and K10 interactions**

**Methods**

The mixed-effects regressions were repeated and compared across three scenarios: 1) using all interviews and not including a K10 variable; 2) using only interviews at which K10 questionnaires were administered but not including a K10 variable; and 3) using only interviews at which K10 questionnaires were administered and including a dichotomous psychological distress variable (K10 score <27 or K10 score >=27).

**Results**

All three scenarios produced qualitatively similar results. The exceptions in terms of statistical significance were that BBV-TRAQ score and attending an ED in the past month were statistically significant on the full set of interviews, but not when the data was restricted to interviews with K10 questionnaires asked, and conversely that changing drug used most from heroin to other and education level were statistically significant when the data was restricted to interviews with K10 questionnaires asked but not on the full dataset (Table S1).

Table A: Linear mixed-effects regression model: using the complete dataset; and using only interviews at which the K10 questionnaire was asked, with either no K10 variable or a dichotomous K10 variable (K10<27 or K10>=27).

| **PWI** Range:  0 (poorest) - 100 (healthiest) | ***Complete dataset (no K10 variable)***  ***(N=2,344)*** | | | ***Interviews with K10 asked, no K10 in regression***  ***(N=1,416)*** | | | ***Dichotomous K10 score indicating likely mental health condition***  ***(N=1,416)*** | | |
| --- | --- | --- | --- | --- | --- | --- | --- | --- | --- |
|  | ***Adjusted coefficient*** | ***95%CI*** | ***p-value*** | ***Adjusted coefficient*** | ***95%CI*** | ***p-value*** | ***Adjusted coefficient*** | ***95%CI*** | ***p-value*** |
| ***Temporal variables*** |  |  |  |  |  |  |  |  |  |
| K10 score >=27 (vs. no) |  |  |  |  |  |  |  |  |  |
| Yes |  |  |  |  |  |  | -14.92*** | (-16.91, -12.93) | **<0.001** |
| Time in study | -0.35 | (-0.99, 0.29) | 0.279 | -0.01 | (-0.76, 0.73) | 0.969 | 0.06 | (-0.63, 0.74) | 0.871 |
| Main income source (vs. wage or salary) |  |  |  |  |  |  |  |  |  |
| Government allowance | -2.31 | (-5.01, 0.40) | 0.095 | -1.18 | (-4.43, 2.07) | 0.476 | -1.16 | (-4.20, 1.89) | 0.457 |
| Other | -2.37 | (-6.15, 1.41) | 0.219 | -1.73 | (-6.11, 2.64) | 0.438 | -2.06 | (-6.11, 1.99) | 0.318 |
| Employed (vs. no) |  |  |  |  |  |  |  |  |  |
| Yes | 3.90*** | (1.86, 5.94) | **<0.001** | 3.50** | (1.05, 5.95) | **<0.01** | 2.57* | (0.35, 4.79) | **<0.05** |
| Current accommodation type (vs. Owner-occupied) |  |  |  |  |  |  |  |  |  |
| Private rental | 0.17 | (-1.99, 2.34) | 0.875 | -3.35* | (-6.17, -0.53) | **<0.05** | -2.39 | (-4.89, 0.11) | 0.061 |
| Public housing | 0.94 | (-1.51, 3.39) | 0.453 | -1.66 | (-4.97, 1.65) | 0.325 | -1.16 | (-4.20, 1.88) | 0.455 |
| Unstable | -4.09** | (-6.65, -1.53) | **<0.01** | -6.20*** | (-9.65, -2.75) | **<0.001** | -5.39*** | (-8.47, -2.31) | **0.001** |
| Drug used most in the past month (vs. heroin) |  |  |  |  |  |  |  |  |  |
| Methamphetamine | -0.96 | (-4.13, 2.20) | 0.550 | -0.97 | (-4.65, 2.72) | 0.606 | 0.23 | (-3.22, 3.69) | 0.894 |
| Cannabis | 1.86 | (-0.01, 3.73) | 0.051 | 1.92 | (-0.54, 4.38) | 0.126 | 2.20 | (-0.05, 4.45) | 0.056 |
| Other | 0.73 | (-1.25, 2.71) | 0.470 | 1.42 | (-1.15, 3.98) | 0.278 | 2.87* | (0.49, 5.25) | **<0.05** |
| AUDIT C (vs. 0) |  |  |  |  |  |  |  |  |  |
| 1-7 | -0.98 | (-2.64, 0.68) | 0.249 | -1.14 | (-3.21, 0.93) | 0.280 | -0.76 | (-2.74, 1.23) | 0.454 |
| >=8 | 0.03 | (-2.02, 2.07) | 0.978 | 0.34 | (-2.16, 2.84) | 0.792 | 1.16 | (-1.20, 3.53) | 0.336 |
| Total injections in the past week | -0.06 | (-0.13, 0.02) | 0.123 | -0.08 | (-0.19, 0.02) | 0.130 | -0.05 | (-0.14, 0.05) | 0.368 |
| Inject more than usual in the past 6 months (vs. no) |  |  |  |  |  |  |  |  |  |
| Yes | -2.57*** | (-3.98, -1.15) | **<0.001** | -2.69** | (-4.60, -0.79) | **<0.01** | -2.14* | (-3.97, -0.31) | **<0.05** |
| Use alone more than 80% of the time (vs. no) |  |  |  |  |  |  |  |  |  |
| Yes | -0.55 | (-2.03, 0.92) | 0.462 | -0.59 | (-2.49, 1.30) | 0.539 | -0.49 | (-2.18, 1.20) | 0.570 |
| BBV-TRAQ-SV score | -0.08** | (-0.13, -0.03) | **0.001** | -0.07 | (-0.14, 0.00) | 0.051 | -0.06 | (-0.13, 0.00) | 0.069 |
| Currently on OST (vs. no) |  |  |  |  |  |  |  |  |  |
| Yes | 1.09 | (-0.59, 2.77) | 0.203 | 1.40 | (-0.74, 3.54) | 0.201 | 1.60 | (-0.24, 3.44) | 0.087 |
| Attended a GP in the past month (vs. no) |  |  |  |  |  |  |  |  |  |
| Yes | -0.98 | (-2.49, 0.53) | 0.204 | 0.04 | (-1.85, 1.94) | 0.964 | 0.47 | (-1.35, 2.29) | 0.612 |
| Any mental health assistance in the past month (vs. no) |  |  |  |  |  |  |  |  |  |
| Yes | -0.33 | (-1.74, 1.09) | 0.652 | 0.02 | (-1.84, 1.89) | 0.980 | 0.90 | (-0.86, 2.65) | 0.316 |
| Attended ED in past month (vs. no) |  |  |  |  |  |  |  |  |  |
| Yes | -2.46* | (-4.82, -0.11) | **<0.05** | -1.36 | (-4.12, 1.39) | 0.331 | -0.69 | (-3.35, 1.97) | 0.612 |
| Heroin overdose in the past six months (vs. no) |  |  |  |  |  |  |  |  |  |
| Yes | -1.47 | (-3.79, 0.86) | 0.217 | -2.13 | (-5.24, 0.98) | 0.179 | -1.18 | (-4.17, 1.8) | 0.437 |
| Intentional overdose in the past12 months (vs. no) |  |  |  |  |  |  |  |  |  |
| Yes | -5.56** | (-9.72, -1.4) | **<0.01** | -10.30*** | (-14.79, -5.81) | **<0.001** | -7.24** | (-11.65, -2.83) | **0.001** |
| Assault victim in past six months (vs. no) |  |  |  |  |  |  |  |  |  |
| Yes | -5.51*** | (-7.24, -3.78) | **<0.001** | -5.65*** | (-7.84, -3.46) | **<0.001** | -3.66*** | (-5.63, -1.69) | **<0.001** |
| Arrested in the past 12 months (vs. no) |  |  |  |  |  |  |  |  |  |
| Yes | -0.58 | (-2.11, 0.95) | 0.455 | 0.47 | (-1.48, 2.42) | 0.637 | 0.23 | (-1.54, 1.99) | 0.801 |
|  |  |  |  |  |  |  |  |  |  |
| ***Stable variables*** |  |  |  |  |  |  |  |  |  |
| Sex (vs. female) |  |  |  |  |  |  |  |  |  |
| Male | -0.70 | (-3.09, 1.70) | 0.569 | -0.30 | (-3.12, 2.52) | 0.834 | -1.43 | (-3.91, 1.04) | 0.257 |
| Age at interview | -0.19 | (-0.38, 0.01) | 0.066 | -0.06 | (-0.29, 0.18) | 0.641 | -0.10 | (-0.31, 0.10) | 0.323 |
| Recruitment site (vs. Inner West) |  |  |  |  |  |  |  |  |  |
| Central | 1.60 | (-1.49, 4.70) | 0.310 | 3.13 | (-1.49, 7.75) | 0.184 | 2.26 | (-1.68, 6.20) | 0.261 |
| Outer-Urban | 0.50 | (-2.56, 3.55) | 0.751 | 3.75 | (-0.88, 8.39) | 0.113 | 2.36 | (-1.74, 6.45) | 0.259 |
| Country of birth (vs. outside of Australia) |  |  |  |  |  |  |  |  |  |
| Australia | 0.04 | (-2.98, 3.06) | 0.979 | 0.50 | (-3.48, 4.48) | 0.806 | 0.71 | (-2.63, 4.05) | 0.676 |
| Speak a language other than English (vs. no) |  |  |  |  |  |  |  |  |  |
| Yes | 0.08 | (-3.68, 3.85) | 0.965 | 0.47 | (-4.43, 5.37) | 0.851 | 0.21 | (-3.85, 4.28) | 0.918 |
| Education (vs. <year 10) |  |  |  |  |  |  |  |  |  |
| Year 10-11 | 1.84 | (-0.70, 4.38) | 0.156 | 2.97 | (-0.54, 6.49) | 0.097 | 2.63 | (-0.41, 5.66) | 0.090 |
| Year 12 or higher | 1.47 | (-1.17, 4.12) | 0.275 | 4.11* | (0.71, 7.51) | 0.018 | 3.12* | (0.12, 6.13) | **<0.05** |
| Duration of injecting career (years) | 0.00 | (-0.01, 0.00) | 0.557 | 0.00 | (-0.01, 0.01) | 0.975 | 0.01 | (0.00, 0.01) | 0.209 |
| Incarceration history (vs. never) |  |  |  |  |  |  |  |  |  |
| Once | 0.01 | (-2.42, 2.43) | 0.995 | -0.47 | (-3.70, 2.76) | 0.776 | -1.04 | (-3.92, 1.83) | 0.478 |
| Twice | 0.55 | (-2.42, 3.51) | 0.717 | 1.19 | (-2.62, 5.01) | 0.539 | 0.68 | (-2.59, 3.95) | 0.682 |
| Three or more times | 1.34 | (-1.57, 4.25) | 0.366 | 0.43 | (-3.15, 4.01) | 0.815 | -0.06 | (-3.16, 3.03) | 0.968 |
|  |  |  |  |  |  |  |  |  |  |
| ***Constant*** | 63.97*** | (50.40, 77.53) | **<0.001** | 60.03*** | (47.95, 72.11) | **<0.001** | 63.68*** | (52.99, 74.38) | **<0.001** |
|  |  |  |  |  |  |  |  |  |  |
| ***Participant random-effect term*** |  |  |  |  |  |  |  |  |  |
| Standard deviation | 11.47 | (10.52, 12.52) |  | 12.26 | (11.07, 13.57) |  | 10.20 | (9.09, 11.45) |  |
|  |  |  |  |  |  |  |  |  |  |
| ***Residual (error term)*** |  |  |  |  |  |  |  |  |  |
| Standard deviation | 13.49 | (12.91, 14.10) |  | 12.72 | (12.03, 13.45) |  | 12.09 | (11.43, 12.79) |  |
| **p* < 0.05, ***p* < 0.01, ****p* < 0.001 |  |  |  |  |  |  |  |  |  |

**The Melbourne Injecting Drug User Cohort Study (MIX) description**

Recruitment for the Melbourne Injecting Drug User Cohort Study (MIX) occurred between November 2008 and March 2010 via three methods: respondent driven sampling (RDS), street outreach and snowball sampling (Horyniak et al., 2013). RDS involved recruiting a small number of ‘seed’ participants, who were given monetary incentives to facilitate recruitment through their own social networks. Street outreach was facilitated by a team of researchers to regularly visited recruitment locations, and eligible participants were recruited by word of mouth and flyers posted in the neighbourhoods. Snowballing was achieved by encouraging participants to invite their own contacts to the study. Participant were eligible if they were between 18 and 30 years old, had injected heroin or methamphetamine at least six times over the past six months, and were currently living in Melbourne. Initially, participants on OST were excluded from the study; however, this criterion was relaxed due to the high number of otherwise eligible participants being excluded.

Experienced fieldworkers conduct face-to-face interviews with participants approximately annually. Attempts to contact participants and schedule interviews commence two months before their due date (annually from baseline date) and, where contact is difficult (e.g. no phone, new address, incarceration), participants are prioritised as they become overdue. Following overdue interviews, further follow-up interviews may be conducted after a minimum of six months to catch up to original due dates.

MIX was approved by the Victorian Department of Human Services (now Department of Health) and Monash University Human Research Ethics Committees. Written informed consent was obtained from all participants.

Horyniak D, Higgs P, Jenkinson R, Degenhardt L, Stoove M, Kerr T, et al. Establishing the Melbourne Injecting Drug User Cohort Study (MIX): rationale, methods, and baseline and twelve-month follow-up results. *Harm reduction journal*. 2013; 10:11.

**Networks II description**

Networks II was a prospective cohort study of the hepatitis C virus in PWID; it combined molecular epidemiology and social network approaches (Aitken et al., 2008). Between July 2005 and 2007, current PWID (who reported injecting at least monthly over the past six months) were recruited from three major street drug markets located across metropolitan Melbourne. Participants were asked to describe the relationships between themselves and their injecting partners and introduce their partners to our field researchers. PWID aged 25 years or younger or who tested anti-HCV and HCV RNA-negative were preferentially recruited for follow-up, and participants were bled and interviewed about their risk behaviour at approximately three-month intervals.

In 2011, participants from the Networks II study (N=69) were rolled into the MIX study, and subsequently interviewed annually with the MIX questionnaire. Their first interviews were labelled as baseline, with follow-up interviews occurring annually as per the MIX protocol. Interview timings are shown in Fig S1.

Aitken, C.K., Lewis, J., Tracy, S.L., Spelman, T., Bowden, D.S., Bharadwaj, M., Drummer, H. and Hellard, M., 2008. High incidence of hepatitis C virus reinfection in a cohort of injecting drug users. *Hepatology*, *48*(6), 1746-1752.


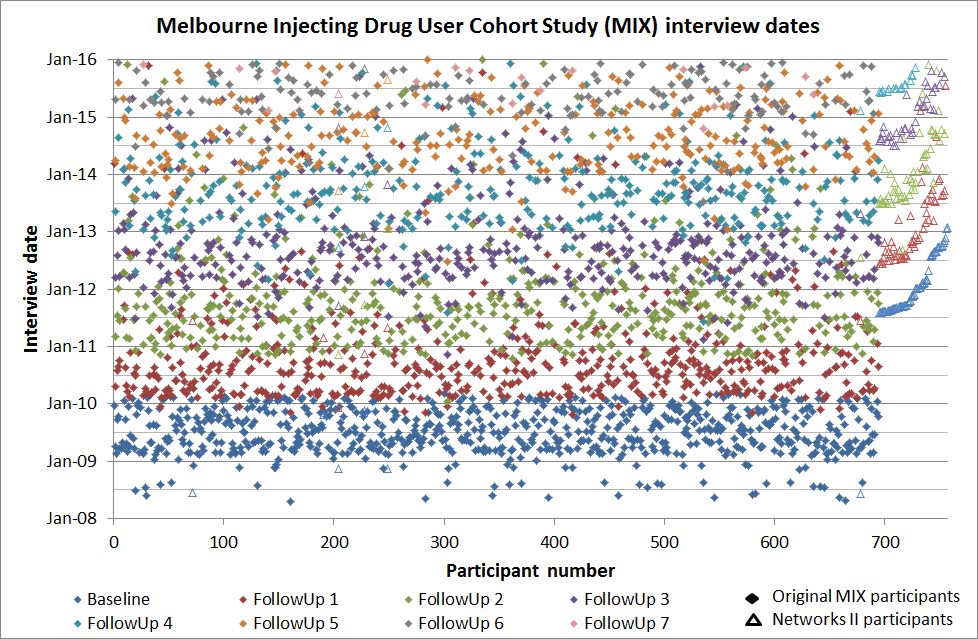


**Fig A: interview dates.** Between 2008 and 2016 interviews have occurred approximately annually for each participant, and are conducted regularly throughout the year.

**The Melbourne Injecting Drug User Cohort Study versus Networks II participant characteristics**

Table B: comparison of the Melbourne Injecting Drug User Cohort Study (MIX) and Networks II characteristics at baseline, stable variables.

|  | **Networks II**  **(N=69)** | | **MIX**  **(N=688)** | | | **p-value (Chi-squared or one-way ANOVA)** | | | **Total**  **(N=757)** | |
| --- | --- | --- | --- | --- | --- | --- | --- | --- | --- | --- |
|  | No. | (%) | | No. | (%) | |  | No. | | (%) |
| Sex |  |  | |  |  | | 0.461 |  | |  |
| Female | 26 | (38%) | | 229 | (33%) | |  | 255 | | (34%) |
| Male | 43 | (62%) | | 459 | (67%) | |  | 502 | | (66%) |
| Median age at recruitment (IQR) | 27.6 | (24.3 – 29.6) | | 34.9 | (31.3 – 40.1) | | <0.001 | 27.9 | | (24.6 – 30.0) |
| Mean age at recruitment (+/- 2 standard errors) | 26.9 | (26.6 - 27.2) | | 35.5 | (33.7 - 37.2) | | <0.001 | 27.6 | | (27.3 - 28.0) |
|  | SD=3.6,  range= 16.8-40.1 | | | SD=7.3,  range= 20.8-52.0 | | |  | SD=4.8,  range= 16.8-52.0 | | |
| Recruitment site |  |  | |  |  | | <0.01 |  | |  |
| Inner West | 46 | (67%) | | 361 | (52%) | |  | 407 | | (54%) |
| Central | 6 | (9%) | | 177 | (26%) | |  | 183 | | (24%) |
| Outer-Urban | 17 | (25%) | | 150 | (22%) | |  | 167 | | (22%) |
| Country of birth |  |  | |  |  | | <0.05 |  | |  |
| Other | 21 | (31%) | | 138 | (20%) | |  | 159 | | (21%) |
| Australia | 46 | (69%) | | 550 | (80%) | |  | 596 | | (79%) |
| Speak a language other than English |  |  | |  |  | | 0.137 |  | |  |
| No | 52 | (78%) | | 582 | (85%) | |  | 634 | | (84%) |
| Yes | 15 | (22%) | | 106 | (15%) | |  | 121 | | (16%) |
| Education |  |  | |  |  | | 0.322 |  | |  |
| Less than year 10 | 20 | (30%) | | 231 | (34%) | |  | 251 | | (33%) |
| Year 10-11 | 28 | (42%) | | 316 | (46%) | |  | 344 | | (46%) |
| Year 12 or higher | 19 | (28%) | | 141 | (20%) | |  | 160 | | (21%) |
| Median length of injecting career (IQR) | 10.2 | (6.2 – 12.7) | | 16.7 | (12.7 – 22.1) | | <0.001 | 10.4 | | (6.7 – 13.4) |
| Incarceration history |  |  | |  |  | | 0.074 |  | |  |
| None | 19 | (29%) | | 277 | (40%) | |  | 296 | | (39%) |
| Once | 18 | (27%) | | 210 | (31%) | |  | 228 | | (30%) |
| Twice | 13 | (20%) | | 91 | (13%) | |  | 104 | | (14%) |
| Three or more times | 16 | (24%) | | 106 | (15%) | |  | 122 | | (16%) |

Table C: comparison of the Melbourne Injecting Drug User Cohort Study (MIX) and Networks II characteristics at baseline, temporal variables.

|  | **Networks II**  **(N=69)** | | **MIX**  **(N=688)** | | **p-value (Chi-squared or one-way ANOVA)** | **Total**  **(N=757)** | |
| --- | --- | --- | --- | --- | --- | --- | --- |
|  | No. | (%) | No. | (%) |  | No. | (%) |
| Mean PWI score (+/- 2 standard errors) | 57.6 | (52.7 - 62.5) | 54.6 | (54.2 - 57.1) | 0.761 | 54.9 | (53.5 – 56.3) |
| Main income source |  |  |  |  | 0.105 |  |  |
| Wage or salary | 11 | (16%) | 59 | (9%) |  | 70 | (9%) |
| Government allowance | 52 | (78%) | 588 | (86%) |  | 640 | (85%) |
| Other | 4 | (6%) | 38 | (6%) |  | 42 | (6%) |
| Employed |  |  |  |  | <0.05 |  |  |
| No | 50 | (75%) | 590 | (86%) |  | 640 | (85%) |
| Yes | 17 | (25%) | 97 | (14%) |  | 114 | (15%) |
| Current accommodation type |  |  |  |  | 0.126 |  |  |
| Owner-occupied | 8 | (12%) | 155 | (23%) |  | 163 | (22%) |
| Private Rental | 28 | (42%) | 214 | (31%) |  | 242 | (32%) |
| Public Housing | 20 | (30%) | 185 | (27%) |  | 205 | (27%) |
| Other | 11 | (16%) | 130 | (19%) |  | 141 | (19%) |
| Drug used the most in previous month |  |  |  |  | <0.01 |  |  |
| Heroin | 26 | (40%) | 415 | (61%) |  | 441 | (59%) |
| Methamphetamine | 2 | (3%) | 43 | (6%) |  | 45 | (6%) |
| Cannabis | 22 | (34%) | 127 | (19%) |  | 149 | (20%) |
| Other | 15 | (23%) | 101 | (15%) |  | 116 | (15%) |
| AUDIT C scores |  |  |  |  | 0.622 |  |  |
| abstinent | 27 | (41%) | 248 | (36%) |  | 275 | (37%) |
| <8 | 22 | (33%) | 269 | (39%) |  | 291 | (39%) |
| >=8 | 17 | (26%) | 168 | (25%) |  | 185 | (25%) |
| Mean total injections in the past week (+/- 2 standard errors) | 8.9 | (7.9 – 9.9) | 9.3 | (6.1 – 12.5) | 0.600 | 8.9 | (8.0 – 9.8) |
| Inject more than usual in the past month |  |  |  |  | 0.083 |  |  |
| No | 44 | (67%) | 359 | (52%) |  | 403 | (54%) |
| Yes | 22 | (33%) | 325 | (47%) |  | 347 | (46%) |
| Use alone more than 80% of the time |  |  |  |  | 0.072 |  |  |
| No | 48 | (76%) | 443 | (65%) |  | 491 | (66%) |
| Yes | 15 | (24%) | 239 | (35%) |  | 254 | (34%) |
| Median BBV-TRAQ score | 6.4 | (5.2 – 7.6) | 6.1 | (2.2 – 10.0) | 0.703 | 6.4 | (5.3 – 7.5) |
| OST status |  |  |  |  | <0.001 |  |  |
| No | 26 | (38%) | 446 | (65%) |  | 472 | (62%) |
| Yes | 43 | (62%) | 242 | (35%) |  | 285 | (38%) |
| Attend GP past month |  |  |  |  | 0.001 |  |  |
| No | 13 | (20%) | 284 | (42%) |  | 297 | (40%) |
| Yes | 53 | (80%) | 400 | (58%) |  | 453 | (60%) |
| Attended psychiatrist/psychologist/social worker/drug counsellor in the past month |  |  |  |  | 0.438 |  |  |
| No | 43 | (65%) | 411 | (60%) |  | 454 | (61%) |
| Yes | 23 | (35%) | 271 | (40%) |  | 294 | (39%) |
| Attend an ED past month |  |  |  |  | <0.05 |  |  |
| No | 63 | (95%) | 590 | (87%) |  | 653 | (87%) |
| Yes | 3 | (5%) | 92 | (13%) |  | 95 | (13%) |
| Heroin overdose in the past six months |  |  |  |  | 0.319 |  |  |
| No | 62 | (94%) | 615 | (90%) |  | 677 | (91%) |
| Yes | 4 | (6%) | 67 | (10%) |  | 71 | (9%) |
| Victim of assault in the past 6 months |  |  |  |  | <0.01 |  |  |
| No | 44 | (83%) | 273 | (64%) |  | 317 | (66%) |
| Yes | 9 | (17%) | 155 | (36%) |  | 164 | (34%) |
| Arrested in the past 12 months |  |  |  |  | 0.001 |  |  |
| No | 44 | (67%) | 313 | (46%) |  | 357 | (48%) |
| Yes | 22 | (33%) | 368 | (54%) |  | 390 | (52%) |

**Comparison of characteristics (combined MIX + Networks II data) by gender**

Table D: comparison of baseline stable variables by gender.

|  | **Women**  **(N=255)** | | | **Men**  **(N=502)** | | | **p-value (Chi-squared or one-way ANOVA)** | | **Total** | |
| --- | --- | --- | --- | --- | --- | --- | --- | --- | --- | --- |
|  | No. | (%) | No. | | (%) |  | | No. | | (%) |
| Median age at recruitment (IQR) | 27.4 | (23.8 – 29.5) | 28.2 | | (25.1 – 30.1) | <0.001 | | 27.9 | | 24.6 – 30.0) |
| Mean age at recruitment (+/- 2 standard errors) | 27.2 | (26.5 - 27.8) | 27.9 | | (27.5 – 28.3) | <0.001 | | 27.6 | | (27.3 – 28.0) |
|  | SD=5.4, range=16.8-52.0 | | SD=4.4, range=18.1-48.8 | | |  | | SD=4.8, range=16.8-52.0 | | |
| Recruitment site |  |  |  | |  | 0.929 | |  | |  |
| Inner West | 137 | (54%) | 270 | | (54%) |  | | 407 | | (54%) |
| Central | 60 | (24%) | 123 | | (25%) |  | | 183 | | (24%) |
| Outer-Urban | 58 | (23%) | 109 | | (22%) |  | | 167 | | (22%) |
| Country of birth |  |  |  | |  | <0.001 | |  | |  |
| Other | 33 | (13%) | 126 | | (25%) |  | | 159 | | (21%) |
| Australia | 220 | (87%) | 376 | | (75%) |  | | 596 | | (79%) |
| Speak a language other than English |  |  |  | |  | <0.001 | |  | |  |
| No | 236 | (93%) | 398 | | (79%) |  | | 634 | | (84%) |
| Yes | 17 | (7%) | 104 | | (21%) |  | | 121 | | (16%) |
| Education |  |  |  | |  | 0.095 | |  | |  |
| Less than year 10 | 73 | (29%) | 178 | | (35%) |  | | 251 | | (33%) |
| Year 10-11 | 117 | (46%) | 227 | | (45%) |  | | 344 | | (46%) |
| Year 12 or higher | 63 | (25%) | 97 | | (19%) |  | | 160 | | (21%) |
| Median length of injecting career (IQR) | 10.0 | (6.1 – 12.9) | 10.6 | | (7.0 – 13.6) | <0.001 | | 502 | | (66%) |
| Incarceration history |  |  |  | |  | <0.001 | |  | |  |
| None | 135 | (54%) | 161 | | (32%) |  | | 296 | | (39%) |
| Once | 68 | (27%) | 160 | | (32%) |  | | 228 | | (30%) |
| Twice | 22 | (9%) | 82 | | (16%) |  | | 104 | | (14%) |
| Three or more times | 25 | (10%) | 97 | | (19%) |  | | 122 | | (16%) |

**Cohort characteristics (combined MIX + Networks II data) at each interview wave**

Table E: temporal variable characteristics for the baseline and first four follow-up interview waves.

|  | **Baseline**  **(N=757)** | | **Follow-up 1**  **(N=584)** | | **Follow-up 2**  **(N=510)** | | **Follow-up 3**  **(N=432)** | | **Follow-up 4**  **(N=328)** | |
| --- | --- | --- | --- | --- | --- | --- | --- | --- | --- | --- |
|  | No. | (%) | No. | (%) | No. | (%) | No. | (%) | No. | (%) |
| Median days from previous interview (IQR) |  |  | 366 | (322 – 512) | 365 | (310 – 420) | 371 | (310 – 413) | 370 | (336 – 427) |
| Mean PWI score (+/- 2 standard errors) | 54.9 | (53.5 – 56.3) | 56.7 | (55.1 – 58.3) | 55.8 | (54.1 – 57.5) | 56.3 | (54.5 – 58.1) | 56.3 | (54.1 – 58.5) |
| Grouped K10 score |  |  |  |  |  |  |  |  |  |  |
| Low | 15 | (24%) | 48 | (23%) | 128 | (26%) | 126 | (29%) | 89 | (27%) |
| Moderate | 19 | (31%) | 49 | (24%) | 103 | (21%) | 111 | (26%) | 78 | (24%) |
| High | 16 | (26%) | 60 | (29%) | 130 | (26%) | 97 | (23%) | 81 | (25%) |
| Very high | 12 | (19%) | 48 | (23%) | 135 | (27%) | 94 | (22%) | 80 | (24%) |
| Main income source |  |  |  |  |  |  |  |  |  |  |
| Wage or salary | 70 | (9%) | 78 | (13%) | 79 | (15%) | 67 | (16%) | 51 | (16%) |
| Government allowance | 640 | (85%) | 468 | (80%) | 402 | (79%) | 344 | (80%) | 255 | (78%) |
| Other | 42 | (6%) | 36 | (6%) | 29 | (6%) | 21 | (5%) | 21 | (6%) |
| Employed |  |  |  |  |  |  |  |  |  |  |
| No | 640 | (85%) | 463 | (79%) | 392 | (77%) | 312 | (72%) | 224 | (68%) |
| Yes | 114 | (15%) | 121 | (21%) | 118 | (23%) | 120 | (28%) | 104 | (32%) |
| Current accommodation type |  |  |  |  |  |  |  |  |  |  |
| Owner-occupied | 163 | (22%) | 122 | (21%) | 81 | (16%) | 54 | (13%) | 29 | (9%) |
| Private Rental | 242 | (32%) | 205 | (35%) | 202 | (40%) | 192 | (44%) | 163 | (50%) |
| Public Housing | 205 | (27%) | 171 | (29%) | 148 | (29%) | 125 | (29%) | 81 | (25%) |
| Other | 141 | (19%) | 85 | (15%) | 79 | (15%) | 61 | (14%) | 55 | (17%) |
| Drug used the most in previous month |  |  |  |  |  |  |  |  |  |  |
| Heroin | 441 | (59%) | 235 | (40%) | 172 | (34%) | 133 | (31%) | 93 | (28%) |
| Methamphetamine | 45 | (6%) | 19 | (3%) | 29 | (6%) | 37 | (9%) | 22 | (7%) |
| Cannabis | 149 | (20%) | 159 | (27%) | 160 | (31%) | 152 | (35%) | 90 | (27%) |
| Other | 116 | (15%) | 151 | (26%) | 131 | (26%) | 89 | (21%) | 107 | (33%) |
| AUDIT C scores |  |  |  |  |  |  |  |  |  |  |
| abstinent | 275 | (37%) | 205 | (35%) | 170 | (33%) | 150 | (35%) | 124 | (38%) |
| <8 | 291 | (39%) | 235 | (40%) | 214 | (42%) | 172 | (40%) | 112 | (34%) |
| >=8 | 185 | (25%) | 141 | (24%) | 124 | (24%) | 110 | (25%) | 92 | (28%) |
| Mean total injections in the past week (+/- 2 standard errors) | 8.9 | (8 – 9.8) | 5.9 | (5.1 – 6.6) | 5.4 | (4.6 – 6.3) | 5.2 | (4.4 – 6.1) | 5.8 | (4.7 – 6.9) |
| Inject more than usual in the past month |  |  |  |  |  |  |  |  |  |  |
| No | 403 | (54%) | 384 | (66%) | 351 | (69%) | 318 | (74%) | 244 | (74%) |
| Yes | 347 | (46%) | 185 | (32%) | 152 | (30%) | 107 | (25%) | 81 | (25%) |
| Use alone more than 80% of the time |  |  |  |  |  |  |  |  |  |  |
| No | 491 | (66%) | 347 | (70%) | 269 | (64%) | 245 | (70%) | 181 | (69%) |
| Yes | 254 | (34%) | 146 | (30%) | 154 | (36%) | 105 | (30%) | 82 | (31%) |
| Mean BBV-TRAQ score (+/- 2 standard errors) | 6.4 | (5.3 – 7.5) | 4.6 | (3.4 – 5.7) | 4.2 | (3 – 5.4) | 2.7 | (1.8 – 3.6) | 3.4 | (2.1 – 4.7) |
| OST status |  |  |  |  |  |  |  |  |  |  |
| No | 472 | (62%) | 275 | (47%) | 205 | (40%) | 170 | (39%) | 130 | (40%) |
| Yes | 285 | (38%) | 309 | (53%) | 305 | (60%) | 262 | (61%) | 198 | (60%) |
| Attend GP past month |  |  |  |  |  |  |  |  |  |  |
| No | 297 | (40%) | 187 | (32%) | 142 | (28%) | 134 | (31%) | 107 | (33%) |
| Yes | 453 | (60%) | 396 | (68%) | 368 | (72%) | 298 | (69%) | 221 | (67%) |
| Attended psychiatrist/psychologist/social worker/drug counsellor in the past month |  |  |  |  |  |  |  |  |  |  |
| No | 454 | (61%) | 373 | (64%) | 346 | (68%) | 305 | (71%) | 232 | (71%) |
| Yes | 294 | (39%) | 210 | (36%) | 164 | (32%) | 127 | (29%) | 96 | (29%) |
| Attend an ED past month |  |  |  |  |  |  |  |  |  |  |
| No | 653 | (87%) | 533 | (91%) | 461 | (90%) | 387 | (90%) | 287 | (88%) |
| Yes | 95 | (13%) | 50 | (9%) | 49 | (10%) | 45 | (10%) | 41 | (13%) |
| Heroin overdose in the past six months |  |  |  |  |  |  |  |  |  |  |
| No | 677 | (91%) | 521 | (89%) | 470 | (92%) | 405 | (94%) | 305 | (93%) |
| Yes | 71 | (9%) | 62 | (11%) | 40 | (8%) | 27 | (6%) | 23 | (7%) |
| Intentional overdose in the past 12 months |  |  |  |  |  |  |  |  |  |  |
| No | 0 | (0%) | 513 | (94%) | 484 | (95%) | 418 | (97%) | 313 | (95%) |
| Yes | 0 | (0%) | 20 | (4%) | 23 | (5%) | 14 | (3%) | 15 | (5%) |
| Victim of assault in the past 6 months |  |  |  |  |  |  |  |  |  |  |
| No | 317 | (42%) | 426 | (73%) | 368 | (72%) | 337 | (78%) | 241 | (73%) |
| Yes | 164 | (22%) | 156 | (27%) | 141 | (28%) | 92 | (21%) | 86 | (26%) |
| Arrested in the past 12 months |  |  |  |  |  |  |  |  |  |  |
| No | 357 | (48%) | 283 | (49%) | 276 | (54%) | 243 | (56%) | 196 | (60%) |
| Yes | 390 | (52%) | 298 | (51%) | 234 | (46%) | 188 | (44%) | 132 | (40%) |

**Loss to follow-up: Comparison of participants with one versus more than one interview**

Table F: comparison of participants with only one interview to those with more than one, stable variables as reported at baseline interviews. Combined MIX + Networks II data.

|  | **Participants with more than one interview**  **(N=585)** | | **Participants with only one interview**  **(N=172)** | | **p-value (Chi-squared or one-way ANOVA)** |
| --- | --- | --- | --- | --- | --- |
|  | No. | (%) | No. | (%) |  |
| Sex |  |  |  |  | <0.05 |
| Female | 211 | (36%) | 44 | (26%) |  |
| Male | 374 | (64%) | 128 | (74%) |  |
| Median age at baseline (IQR) | 28.0 | (24.9 – 30.1) | 27.4 | (24.4 – 29.6) | <0.05 |
| Recruitment site |  |  |  |  | <0.001 |
| Inner West | 298 | (51%) | 109 | (63%) |  |
| Central | 138 | (24%) | 45 | (26%) |  |
| Outer-Urban | 149 | (25%) | 18 | (10%) |  |
| Country of birth |  |  |  |  | <0.01 |
| Other | 108 | (19%) | 51 | (30%) |  |
| Australia | 475 | (81%) | 121 | (70%) |  |
| Speak a language other than English |  |  |  |  | 0.001 |
| No | 503 | (86%) | 131 | (76%) |  |
| Yes | 80 | (14%) | 41 | (24%) |  |
| Education |  |  |  |  | 0.558 |
| Less than year 10 | 188 | (32%) | 63 | (37%) |  |
| Year 10-11 | 269 | (46%) | 75 | (44%) |  |
| Year 12 or higher | 126 | (22%) | 34 | (20%) |  |
| Median length of injecting career at first K10 survey (IQR) | 10.6 | (6.8 – 13.5) | 10.3 | (6.1 – 12.8) | 0.395 |
| Incarceration history |  |  |  |  | 0.827 |
| None | 234 | (40%) | 62 | (36%) |  |
| Once | 173 | (30%) | 55 | (32%) |  |
| Twice | 79 | (14%) | 25 | (15%) |  |
| Three or more times | 94 | (16%) | 28 | (16%) |  |

Table G: comparison of participants with only one interview to those with more than one, temporal variables as reported at baseline. Combined MIX + Networks II data.

|  | **Participants with more than one interview**  **(N=585)** | | **Participants with only one K10 survey**  **(N=172)** | | **p-value (Chi-squared or one-way ANOVA)** |
| --- | --- | --- | --- | --- | --- |
|  | No. | (%) | No. | (%) | p-value |
| Main income source |  |  |  |  | 0.974 |
| Wage or salary | 54 | (9%) | 16 | (9%) |  |
| Government allowance | 493 | (85%) | 147 | (85%) |  |
| Other | 33 | (6%) | 9 | (5%) |  |
| Employed |  |  |  |  | 0.225 |
| No | 489 | (84%) | 151 | (88%) |  |
| Yes | 93 | (16%) | 21 | (12%) |  |
| Current accommodation type |  |  |  |  | 0.411 |
| Owner-occupied | 130 | v22%) | 33 | (19%) |  |
| Private Rental | 178 | (31%) | 64 | (37%) |  |
| Public Housing | 162 | (28%) | 43 | (25%) |  |
| Other | 110 | (19%) | 31 | (18%) |  |
| Drug used the most in previous month |  |  |  |  | 0.063 |
| Heroin | 335 | (58%) | 106 | (62%) |  |
| Methamphetamine | 36 | (6%) | 9 | (5%) |  |
| Cannabis | 126 | (22%) | 23 | (13%) |  |
| Other | 83 | (14%) | 33 | (19%) |  |
| AUDIT C scores |  |  |  |  | <0.05 |
| abstinent | 199 | (34%) | 76 | (44%) |  |
| <8 | 237 | (41%) | 54 | (32%) |  |
| >=8 | 144 | (25%) | 41 | (24%) |  |
| Mean total injections in the past week (+/- 2 standard errors) | 8.4 | (7.5 – 9.4) | 10.5 | (7.9 – 13.2) | 0.058 |
| Inject more than usual in the past month |  |  |  |  | <0.01 |
| No | 294 | (51%) | 109 | (64%) |  |
| Yes | 286 | (49%) | 61 | (36%) |  |
| Use alone more than 80% of the time |  |  |  |  | <0.01 |
| No | 394 | (69%) | 97 | (57%) |  |
| Yes | 181 | (31%) | 73 | (43%) |  |
| Mean BBV-TRAQ score (+/- 2 standard errors) | 7.0 | (5.6 – 8.4) | 4.4 | (2.8 – 6.0) | 0.059 |
| OST status |  |  |  |  | <0.01 |
| No | 349 | (60%) | 123 | (72%) |  |
| Yes | 236 | (40%) | 49 | (28%) |  |
| Attend GP past month |  |  |  |  | <0.05 |
| No | 217 | (37%) | 80 | (47%) |  |
| Yes | 363 | (63%) | 90 | (53%) |  |
| Attended psychiatrist/psychologist/social worker/drug counsellor in the past month |  |  |  |  | 0.299 |
| No | 345 | (60%) | 109 | (64%) |  |
| Yes | 233 | (40%) | 61 | (36%) |  |
| Attend an ED past month |  |  |  |  | 0.248 |
| No | 509 | (88%) | 144 | (85%) |  |
| Yes | 69 | (12%) | 26 | (15%) |  |
| Heroin overdose in the past six months |  |  |  |  | 0.797 |
| No | 524 | (91%) | 153 | (90%) |  |
| Yes | 54 | (9%) | 17 | (10%) |  |
| Victim of assault in the past 6 months |  |  |  |  | 0.221 |
| No | 250 | (43%) | 67 | (39%) |  |
| Yes | 137 | (24%) | 27 | (16%) |  |
| Arrested in the past 12 months |  |  |  |  | 0.174 |
| No | 284 | (49%) | 73 | (43%) |  |
| Yes | 294 | (51%) | 96 | (56%) |  |
